# Characterization of the Biogenic Volatile Organic Compounds (BVOCs) and Analysis of the PR1 Molecular Marker in *Vitis vinifera* L. Inoculated with the Nematode *Xiphinema index*

**DOI:** 10.3390/ijms21124485

**Published:** 2020-06-24

**Authors:** Giulia Castorina, Flaminia Grassi, Gabriella Consonni, Sara Vitalini, Roberto Oberti, Aldo Calcante, Enrico Ferrari, Monica Bononi, Marcello Iriti

**Affiliations:** Department of Agricultural and Environmental Sciences, University of Milan, 20133 Milan, Italy; giulia.castorina@unimi.it (G.C.); flaminia.grassi2@gmail.com (F.G.); gabriella.consonni@unimi.it (G.C.); sara.vitalini@unimi.it (S.V.); roberto.oberti@unimi.it (R.O.); aldo.calcante@unimi.it (A.C.); enrico.ferrari@unimi.it (E.F.); monica.bononi@unimi.it (M.B.)

**Keywords:** BVOCs, dagger nematodes, GC-MS, grapevine, monoterpenes, *PR1* gene, sesquiterpenes, SPME, *Xiphinema index*

## Abstract

Upon pathogen attack, plants very quickly undergo rather complex physico-chemical changes, such as the production of new chemicals or alterations in membrane and cell wall properties, to reduce disease damages. An underestimated threat is represented by root parasitic nematodes. In *Vitis vinifera* L., the nematode *Xiphinema index* is the unique vector of *Grapevine fanleaf virus*, responsible for fanleaf degeneration, one of the most widespread and economically damaging diseases worldwide. The aim of this study was to investigate changes in the emission of biogenic volatile organic compounds (BVOCs) in grapevines attacked by *X. index*. BVOCs play a role in plant defensive mechanisms and are synthetized in response to biotic damages. In our study, the BVOC profile was altered by the nematode feeding process. We found a decrease in β-ocimene and limonene monoterpene emissions, as well as an increase in α-farnesene and α-bergamotene sesquiterpene emissions in nematode-treated plants. Moreover, we evaluated the PR1 gene expression. The transcript level of *PR1* gene was higher in the nematode-wounded roots, while in the leaf tissues it showed a lower expression compared to control grapevines.

## 1. Introduction

The European Union is the world’s main wine producer, with a share of about 60% [1]. Given the economic relevance of *Vitis vinifera* L., grapevine pests are of rising interest to agrochemical companies and plant researchers. Among root parasites, nematodes can go undetected for years, especially in perennial crops, but eventually, they strongly decrease crop productivity.

The phylum Nematoda is largely widespread around the world and occupies a huge range of ecological niches [2]. In soil, nematodes play an important role in the decomposition of organic matter and the recycling of nutrients, determining the health of the soil itself. However, several taxa are harmful to many crops of economic importance [3], such as grapevine.

Annual crop losses caused by plant-parasitic nematodes are estimated at 8.8–14.6% of total crop production and 80 billion USD worldwide [4,5]. At least 2000 species of plant-parasitic nematodes are characterized by the presence of a stylet used for root tissue penetration. Some species are endoparasitic, others ectoparasitic [6]. Worldwide, several grapevine-parasitic nematodes can be mentioned, but root-knot nematodes *Meloidogyne* spp. and dagger nematode *Xiphinema index* are the most diffused. They are representative of the two root-feeding models, endoparasitic and ectoparasitic, respectively. *X. index* is a soil-borne nematode that lives in proximity to the rhizosphere [7] and feeds on cell content thanks to its strong stylet [8]. *X. index* is per se a harmful pathogen for viticulture because it causes root necrosis and deformation which considerably reduce productivity [9]. Besides, it specifically transmits the *Grapevine fanleaf virus* (GFLV) [10,11,12], whose symptoms are belatedly visible at the leaf level. Nevertheless, GFLV disease can lead to severe economic losses with a yield decrease up to 80% [13] due to the reduction in fruit quality and the shortening of plant longevity [14].

Preventive application of nematicides, due to their limited efficacy in pest control and negative impact on the environment, is no longer used routinely by farmers [15]. For this reason, it is of fundamental importance to find a way to detect nematode attacks early and prevent their damage.

Plants defend themselves from parasite attacks in different ways, in continuous coevolution with pathogens [16]. Their stationary status makes them vulnerable but plants limit damage using a variety of defense mechanisms [17], so disease is an exceptional condition rather than normality. Defense mechanisms can be both constitutive and inducible, but while the first is pre-established and energetically irrelevant, the second requires a high amount of energy and is stimulated by pathogen attacks. Inducible defenses act at the time of pathogen recognition and rapidly limit possible damages. A typical feature of resistance is the induction of cell death at the site of attempted attack such as the hypersensitive response (HR) [18], a mechanism which highly limits pathogen proliferation in the host organism. Subsequently, a large set of defense-related genes are expressed as resistance develops [19]. HR settlement involves the induction of many defense mechanisms such as the strengthening of cell walls, salicylic acid (SA) pathway, synthesis of phytoalexins organic molecules and HR-related molecules (H_2_O_2_) which are among the main molecules secreted and produced during the plant/pathogen interaction [20]. Among proteins involved in defense mechanisms, the so-called pathogenesis-related proteins (PRs) certainly have deep importance in plant protection.

Besides accumulating locally in the infected tissues, PRs are also induced systemically, associated with the development of systemic acquired resistance (SAR) against other infections [21]. For example, in *Arabidopsis thaliana* there are 17 evolutionarily conserved families of PRs [22] with 22 *PR1*-type genes [23], but only one of them is activated by pathogens whereas other *PR1*-type genes are constitutively expressed [24].

Nematode attack can affect PR gene expression through the injection of substances produced in salivary glands, which can inhibit host response. Root-knot nematodes secrete molecules called “effectors” to facilitate the invasion of the host roots, avoid plant defense responses and reprogram root cells to form specialized feeding cells [25]. Various PRs have been identified as direct targets of nematode effectors, but nevertheless, their precise mode of action remains largely unknown and only a few of their direct targets in plants have been identified [25].

Plants can either act directly on pathogen feeding and reproduction, for example through trichomes or thorns or indirectly, through the emission of phytochemicals. In particular, the production of secondary metabolites is a defense strategy to cope with several pests [26]. Among secondary metabolites, plants produce root-specific volatile organic compounds (VOCs) [27], which can influence the rhizosphere and plant-pathogen interaction [28,29,30,31].

There is growing evidence that both the quantity and type of volatiles produced by roots are dramatically altered by the presence of different biotic and abiotic stresses [32,33]. It was also reported that VOC changes in response to pathogens or symbionts are species-specific [34]. For example, it has been demonstrated that plants produce chemical signals to ward off herbivorous insects by attracting their natural enemies [35]. Biogenic VOCs (BVOCs) are the major secondary metabolites in plants involved in communications between plants and the external environment, in a mechanism known as “talking plants” [36].

The term BVOCs defines organic atmospheric gases different from carbon dioxide and monoxide [37]. BVOCs include a wide range of different compounds, among which isoprene and monoterpenes are the most prominent [37]. BVOC emission can be stimulated in response to insect feeding [38] and it is largely demonstrated that plants vary the emission of organic compounds in different plant-parasite interactions [16,38,39,40,41]. Moreover, BVOC emission seems to be stimulated by the presence of elicitors present in parasite oral secretions [39].

In this context, we investigated the response of grapevine cuttings to the nematode feeding process through BVOC profiling and *PR1* gene expression, with the aim of exploring the potential of this approach in the detection of an early signal of the nematode attack on the plant root system.

## 2. Results

### 2.1. The BVOC Profile

**Figure 1:**
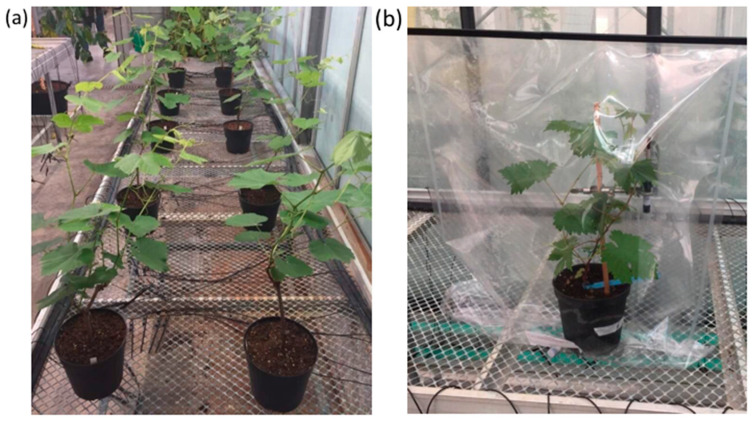
The cuttings of grapevines cv Chardonnay were grown in pots under greenhouse conditions (**a**). Representative image of grapevine in SKC (Tedlar gas sampling) bags to collect BVOCs (biogenic volatile organic compounds) in headspace air through fiber for SPME (solid phase microextraction) sampling technique (**b**).

To examine the effect of the nematode feeding process on the BVOC emission, we measured their profile in nematode wounded (NW) or control (WW) plants over a period of 72 h. All of the grapevine cuttings were grown under greenhouse conditions to avoid influence of environmental factors (Figure 1).

**Figure 2:**
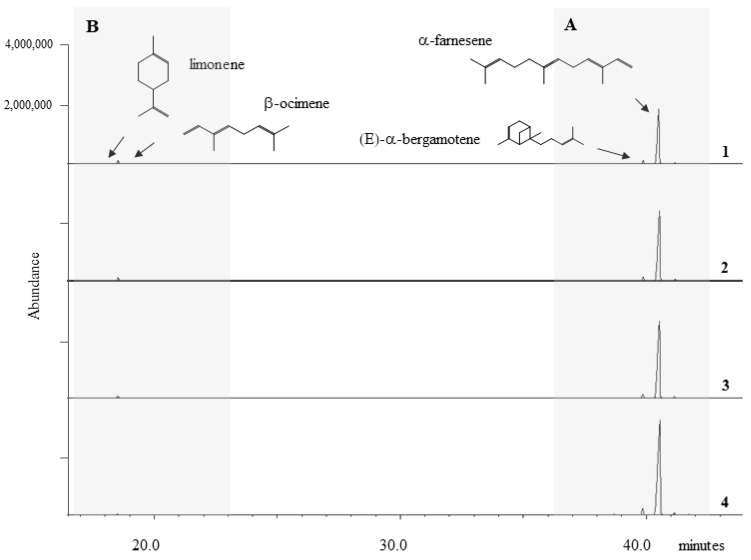
Representative HS-SPME-GC/MS traces from *Vitis vinifera* plants at four different times: (1) before inoculation, (2) 24 h, (3) 48 h and (4) 72 h after inoculation. The grey zone **A** highlights the presence of monoterpenes limonene and β-ocimene, while the grey zone **B** highlights the presence of sesquiterpenes (E)α-bergamotene and α-farnesene.

GC-MS (gas chromatography-mass spectrometry) analysis allowed the identification of the main emitted compounds at different times: before and 24, 48 and 72 h after inoculation. Sesquiterpene and monoterpene biosynthesis pathways, which seemed to be largely involved in grapevine response mechanisms to biotic stress, were easily detectable by SPME (solid phase microextraction) and GC-MS techniques. Although in WW plants, BVOC emission showed a similar trend, in NW plants we observed variations with respect to the pre-inoculation period. Two main classes of volatile compounds exhibited changes in their profile: sesquiterpenes tended to increase while monoterpenes showed decreasing values over time (Figure 2).

All investigated plants emitted a high amount of α-farnesene, the most released compound both in WW and NW plants, during the experimentation period (pre inoculation included), followed by β-ocimene and (*E*)-α-bergamotene. Limonene was the least emitted compound (Figure 2).

#### 2.1.1. Trend in Sesquiterpene Response

**Figure 3:**
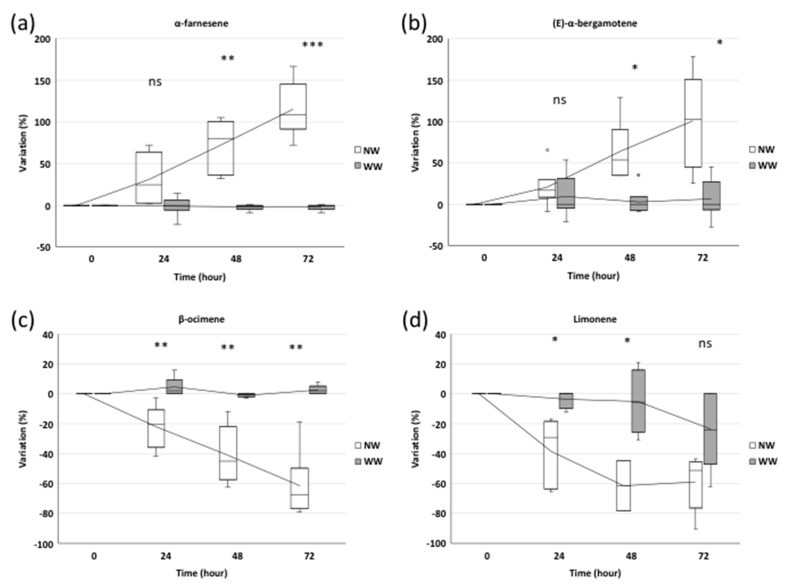
The BVOC profile: Relative amount of the sesquiterpenes α-farnesene (**a**) and (E)-α-bergamotene (**b**) and of the monoterpenes β-ocimene (**c**) and limonene (**d**) evaluated at 0, 24, 48 and 72 h after nematode inoculation in wounded (NW) and without wounding (WW) plants. The box plots refer, for each time point, to three and six independent biological replicates for WW and NW, respectively. A Student’s t-test (df = 7) was applied (*** *p* ≤ 0.001, ** *p* ≤ 0.01, * *p* ≤ 0.05, ns—no statistically significant differences).

The two sesquiterpenes detected by the SPME and GC-MS analysis showed an increase in their emissions after nematode treatments. In particular, 24 h after inoculation, α-farnesene showed a 33% higher emission in NW plants, not significantly different from that of WW plants (Figure 3a). Meanwhile, 48 and 72 h from inoculation, the α-farnesene emission increased by 76% (*p* ≤ 0.01) and 120% (*p* ≤ 0.001), respectively, in NW plants compared to WW ones (Figure 3a).

A similar trend was detected for (*E*)-α-bergamotene. After 24 h, its emission profile in NW plants did not differ from that of WW plants (Figure 3b). After 48 h and 72 h, (*E*)-α-bergamotene showed a 99% and 41% increase, respectively, in NW cuttings compared to WW plants (*p* ≤ 0.05) (Figure 3b).

#### 2.1.2. Trend in Monoterpene Response

Unlike sesquiterpenes, the monoterpene emission profile showed a negative trend in NW plants compared to WW ones. We observed 31%, 39% and 67% reductions in the β-ocimene amount of NW plants compared to WW grapevines, at 24, 48 and 72 h from inoculation, respectively (*p* ≤ 0.01) (Figure 3c).

Similarly, limonene showed significant reductions (−31% and −35%) (*p* ≤ 0.05) after 24 and 48 h in NW samples, while in both NW and WW plants, it was characterized by a similar decreasing emission after 72 h (Figure 3d).

### 2.2. PR1 Genes Expression

**Figure 4:**
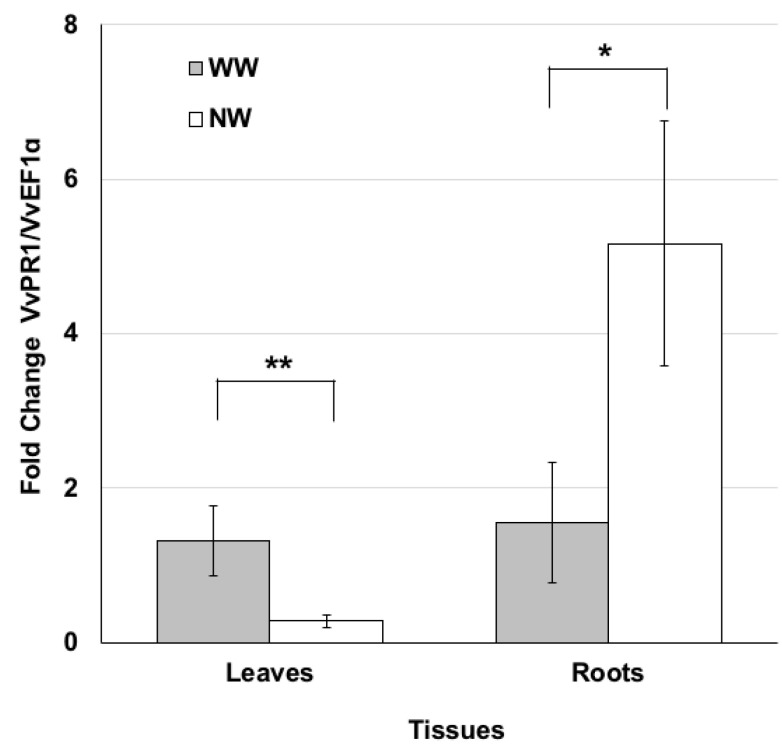
Nematode feeding modulates the expression of the pathogenesis-related protein PR1. Pattern of *VvPR1* transcript accumulation analyzed by real time PCR in the leaf and root tissues of grape plants grown under nematode (NW) or control (WW) conditions for 3 months. Values represent the mean fold change variations ± SD of three and four independent biological replicates for WW and NW, respectively. Significant differences were assessed by Student’s t-test (df = 5) (* *p* < 0.05, ** *p* < 0.01).

PR proteins are defined as plant proteins induced in pathological or related situations [42] and concurring with plant protection. On this basis, the *PR1* gene expression profile in leaf and root tissues was examined three months after nematode inoculation. In particular, we investigated the transcriptomic changes induced by the nematode feeding process. The expression levels of the pathogen-related *VvPR1* gene were lower in the leaves of NW than WW plants (Figure 4), while the expression profile of *VvPR1* analyzed in the roots exhibited an opposite trend. The transcripts accumulated in NW plants compared to WW ones (Figure 4).

Lastly, we assessed the efficiency of our nematodes–plant model experimental system by evaluating the nematode population growth (Table 1). Preliminary pilot experiments with grapevine cuttings revealed a substantial increase in the number of dagger nematodes after three months. Particularly, the initial number of nematodes placed in pots multiplied approximately four times, as reported in Table 1. The increase in the nematode population, which is consistent with an active feeding process on grapevine roots, confirmed that our experimental system was properly functioning.

A small number of nematodes was also recorded in WW plants as well as in plant-free pots, probably indicating the presence of an endemic soil nematode population.

## 3. Discussion

Plants must cope with a plethora of biotic stresses due to attacks from herbivores and pathogens throughout their life cycle [43]. Among grapevine pathogens, nematodes are particularly harmful. When nematodes feed on roots, they profoundly damage them, compromising the plant’s productivity and longevity. During their evolution, plants have developed a variety of different ways to protect themselves from damage, especially the secondary metabolite production. Terpenoids are the most diverse group. They act as phytoalexins in plant direct defense or as signals in indirect defense responses [41]. Therefore, the volatiles released from plants under attack can benefit both the plant, by attracting the herbivorous natural enemies, and the parasitoid, by indicating the presence of a potential host on the plant [44]. The biosynthesis pathways of monoterpenes, diterpenes and sesquiterpenes include the synthesis of the precursor C_5_ isopentenyl diphosphate (IPP) and its allylic isomer dimethylallyl diphosphate (DMAPP), the synthesis of immediate diphosphate precursors, and the formation of different terpenoids [41]. The plant defense responses to herbivores are complex, but the induction of phytohormones jasmonic acid, ethylene, salicylic acid and gene expression are correlated to the different feeding strategies of herbivores and the damage intensity [39].

Plants produce different volatiles at different times of the damaging processes so that it could be possible to distinguish older wounds from new ones according to emitted compounds. The early stages of plant damage are characterized by the release of “green leafy” volatiles ((Z)-3-hexenal, (*Z*)-3-hexenol, (*Z*)-3-hexenyl acetate) and some plant-specific constitutive compounds [44,45]. Older damages are characterized by the higher emission of other volatiles such as (*E*)-β-ocimene, linalool, (*E*)-4,8-dimethyl-1,3,7-nonatriene, (*E*)-β-farnesene, (*E,E*)-α-farnesene and (*E,E*)-4,8,12-trimethyl-1,3,7,11-tridecatetraene [45]. α-Bergamotene, an herbivore-induced compound, also plays a role in plant defense mechanisms, but its daytime emission pattern appears to be largely independent of elicitation time [46].

In this study, BVOCs were collected over 72-h period, which followed nematode infection, and the emission blend was evaluated both in WW plants and NW grapevine cuttings. Samples principally emitted four compounds, namely sesquiterpenes α-farnesene and (*E*)-α-bergamotene and the monoterpenes β-ocimene and limonene. Their release tended to vary 24 h after nematode inoculation in all NW plants, while in WW samples BVOC emission was quite linear. This is in agreement with Paré et al. [38], according to which, during this time, a series of inducible biochemical reactions useful for the BVOC emission occurs [38]. Indeed, it seems that all these compounds are synthesized de novo after a certain period from damage and only in small quantities before stress induction [39].

In our case, α-farnesene and (*E*)-α-bergamotene also started to increase after 24 h from the nematode wounding and their emission continued to significantly grow throughout the experimentation. On the contrary, both β-ocimene and limonene tended to decrease during the experimentation in NW plants, starting from 24 h after inoculation.

Among monoterpenes, β-ocimene is a very common plant volatile released in large amounts from the leaves and flowers of many plant species [47], while limonene is the most widespread terpene in the world [48]. β-Ocimene is known to be emitted in response to herbivore damage [39,45,49] and it can elicit a defense response in neighboring plants [50]. Limonene acts against many insects, mites and microorganisms [51], but it is also involved in abiotic stress protection and particularly can play a role in protecting plants from heat damage, because of its activity in fluidification and membrane stabilization [48]. However, in our study, both compounds showed an inverse trend, decreasing after 24 h from the nematode feeding process, and continuing to decrease for the remaining 48 h.

Among the inducible responses associated with resistance to potential pathogens, there is the synthesis of a wide array of proteins [52], especially PR proteins [42]. They are involved in host-pathogen interactions, being one of the first biotic stress-induced responses [22]. In particular, PR1 proteins are the most studied because they are generally considered as marker proteins for SAR [21,53]. Members of the PR1 family are highly conserved in plants and their homologues have also been found in fungi, insects, and vertebrates, including humans [22]. As an example, in *A. thaliana* only a single *PR1* gene (At2g14610), activated by infections, insect attacks or chemical treatments, relates to pathogen resistance, whereas ten and eight different PR-1-type genes are constitutively expressed in roots and pollen, respectively, contributing to other functions [23,24]. In grapevine, PR1 proteins are also constitutively expressed in callus cultures [54]. Within infected leaves, PRs accumulate both in epidermal and mesophyll cells, as well as in the vascular bundles, glandular trichomes and crystal idioblasts [24].

To estimate the nematode effect in plant response, we analyzed the *PR1* expression in root and foliar tissues three months after nematode inoculation. In general, PR1 proteins were more expressed in roots than in leaves, both in NW and WW plants. Particularly, PR1 proteins were highly expressed in the roots of infected grapevines, unlike in WW plants where they were less stored. On the other hand, NW plants tended to express a smaller quantity of PR1 proteins in foliar tissues than WW plants. Nematodes can alter PR1 protein expression, up- or down-regulating their production in different vegetal organs, altering plant defense response. Hamamouch and colleagues [55] demonstrated that in *A. thaliana* plants, parasitized by *Meloidogyne incognita*, PR1 proteins were highly expressed in roots, while their expression was down-regulated in leaves. Although they are different nematode species, our results seem to be coherent with previous ones.

In conclusion, nematodes are omnipresent and include many plant-parasitic species that can cause enormous economic losses in various crops [56]. Due to the nematode’s harmful action on vineyards yield, it is mandatory to better understand plant-pathogen relationships. Currently, there are few agrochemical options to manage nematode infection and none for GFLV [57,58]. Furthermore, the use of plant material resistant to nematodes is often difficult because of the incompatibility of the rootstock with the grafting material [59,60]. Nowadays, in Europe, all grapevine rootstocks are susceptible to attack by these parasites. In case of GFLV infection, the only solution is the plant’s extirpation and, in the absence of fumigation treatment, the vineyards infested with *X. index* normally require a long fallow period (4–7 years) [61].

Lastly, an understanding of how plants modulate BVOC release in response to the soil-borne parasitic attack, as well as obtaining detailed information on their emission profile, and through the development of new simple and portable sensing devices, based, for example, on olfactometric technology to detect infection presence early, could significantly contribute to improving crop protection.

## 4. Materials and Methods

### 4.1. Plant Material

Nine cuttings of *V. vinifera* (cv. Chardonnay) pruned keeping only one principal shoot grown in pots with peat soil, under controlled conditions, in June 2018. In the greenhouse, the temperature was from a minimum of 18 °C to a maximum of 28 °C and the photoperiod was set to 16 h of light and 8 h of dark. The samples were divided into groups of three grapevine cuttings each. Every group represented an experimental repetition with one control plant (WW) and two plants as independent biological replicates for nematode treatments (NW). A total number of three control (WW) and six inoculated (NW) plants were employed during the experimentation. The experiment was repeated three times, with one WW and two NW plants per group.

### 4.2. Nematode Isolation

Nematodes were isolated from a GFLV-infected vineyard in Puegnago del Garda (Brescia, Italy). Disruptive analyzes were carried out for the nematode species identification and their characterization was conducted considering the morphological and morphometric parameters of adult females, according to Groza and Mezaand [62,63]. The vineyard nematode population was composed of the *X. index* species to a portion of 70% (data not shown).

For our experiment, we provided isolation of vital nematodes by collecting soil samples in the rhizosphere of virus-infected grapevines, at about 20 cm depth, where *X. index* is more active. The soil samples were placed in containers with some paper on the bottom. The soil was watered and water percolated overnight. The paper separated the percolating water containing nematodes from particulate. Collected water samples were observed in 60 mm Petri dishes under an optic microscope (4X) to select the nematodes to be used for inoculations based on their vitality. To evaluate the population growth, at the end of the tests, after three months from inoculation, the nematodes were re-isolated from the total substrate contained in the pots where the grapevine cuttings were grown. The soil percolation system, slightly modified, was partially integrated according to Van Bezooijen [64]. The water containing the nematodes, percolated on the bottom of the containers, was collected with a 5 mL serological pipette and transferred to a 20 mL Falcon, then centrifuged at 1800 g for 4 min. Afterwards, the water was observed under the microscope using a 60 mm Petri dish with a grid on the bottom to facilitate nematode count.

### 4.3. SPME Sampling and GC-MS Analysis

To study the plant BVOC emission caused by the nematode feeding process, pots were enclosed in Tedlar gas sampling bags (SKC, PA, USA) (Figure 1b). Solid phase microextraction (SPME), performed with 2 cm of DVB/CAR/PDMS 50/30 mm (divinylbenzene/carboxen/polydimethylsiloxane) fibers (Supelco, Italy), was applied to analyze BVOCs in the headspace air at different points in time, by sampling at 0, 24, 48 and 72 h after inoculation. The SPME fiber was first conditioned according to the manufacturer’s instructions and then was inserted in the plant bag by avoiding any disturbance of the internal atmosphere. For each time sampling, a fiber was exposed to headspace gas starting from midday and retracted after 24 h. A single SPME fiber was employed for each plant so that at each sampling time, BVOCs were collected and injected in GC-MS once. Afterward, according to conditions previously described [65], the BVOCs absorbed by the fiber were thermally desorbed for 10 min at 240 °C in gas chromatography-mass spectrometry (GC-MS) with a split/splitless injection port, operating in a split mode (1:5). Before every sampling, the fiber was reconditioned for 20 min in the GC injection port at 240 °C, and blank runs were carried out before every analysis.

The GC-MS analyses were carried out using a Shimadzu 2010 gas chromatograph coupled to a Shimadzu QP-2010 MSD quadrupole mass spectrometer (Shimadzu, Italy). A Restek Rxi-5ms 30 m × 0.25 mm, 0.25 µm film thickness capillary silica column (Restek, Italy) was used for the compound separation. The operating conditions were: helium flow 1.0 mL min^−1^ and oven temperature 35 °C for 3 min, increased to 240 °C at a rate of 3 °C min^−1^, and 30 min hold; injection was in split mode (1:5), and the injector and detector temperatures were set at 240 °C and 260 °C, respectively. The MS ran in electron impact (EI) mode was at 70 eV electron energy and the temperature of the ion source was 200 °C. Mass spectra were acquired over the mass range 40–300 a.m.u.. BVOCs were identified by matching their mass spectra with the reference mass spectra of an in-house databank (Di.S.A.A. library) and that of NIST 147 library. The GC-MS analysis resulted in mass spectra graphics, each reporting the relative quantity of released bio-volatiles. The relative quantity was calculated from the area underlying the mass-spectra graphics before and 24, 48 and 72 h after nematode inoculation. To evaluate changes in BVOC emission, normalization of data was applied, using the following formula:

(1) $$Variation=\frac{\left(Area\;T_{n}-Area\;T_{0}\right)}{Area\;T_{0}}$$

where *Area T_n_* represents the BVOC emission at a specific time and *Area T*_0_ represents the basal BVOC emission. In this way, the BVOC emissions before and after the nematode inoculation were compared. Statistical differences were determined using Student’s *t*-test performed with statistical package XLSTAT (Microsoft Excel).

### 4.4. Quantitative Gene Expression Analysis

Total RNA was extracted from both leaf and root tissues using the Rapid CTAB Protocol method by Gambino and colleagues [66] and treated with RQ1 RNase-Free DNase (Promega) according to the manufacturer’s instructions. First-strand complementary DNA (cDNA) was synthetized with the High-Capacity cDNA Reverse Transcription Kit (Applied Biosystems) from 500 ng of total RNA, according to the manufacturer’s instructions. Real-time PCR was performed with the 7300 Real-Time PCR System (Applied Biosystems), using GoTaq qPCR Master Mix (Promega), in a final volume of 10 μL. The following cycle was used: 10 min pre-incubation at 95 °C, followed by 35 cycles of 15 s at 95 °C and 1 min at 60 °C. The relative transcript level of each gene was calculated by the 2^-ΔΔCt^ method [67] using the expression of the *VvEF1a* gene as a reference. Statistical differences were determined using Student’s t-test performed with statistical package XLSTAT (Microsoft Excel). The gene-specific primers VvPR1_F1: GGAGTCCATTAGCACTCCTTTG and VvPR1_R1: CATAATTCTGGGCGTAGGCAG [68] or VvEF1*a*_F1: AACCAAAATATCCGGAGTAAAAGA and VvEF1*a*_R1: GAACTGGGTGCTTGATAGGC [69] were used for the amplification of the *VvPR1* and *VvEF1a* genes, respectively. The gene sequence from this article can be found in the GenBank/EMBL databases under the following NCBI accession numbers: XM_002273752.3 (*VvPR1*) and XM_002284888.3 (*VvEF1a*).

## Figures and Tables

**Table 1:** Nematode population growth. Inoculum density (Inoculation N°) expressed as the number of dagger-nematodes introduced in each pot at the start of the experiment. The without wounding (WW) control plants did not receive the inoculum (-). An inoculum of 80 or 50 nematodes was dispensed to the nematode wounded (NW) grapevines. After 3 months, at the end of the experiment, the numbers of nematodes (Final N°) were analyzed. Three control (WW1-WW3) and six inoculated (NW1-NW6) plants were analyzed.

	WW1	WW2	WW3	NW1	NW2	NW3	NW4	NW5	NW6
**Inoculation N°**	-	-	-	80	80	50	50	80	80
**Final N°**	<20	<20	<20	~320	~320	~200	~138	~184	~110
